# Vaspin, a Compensatory Mechanism Against High Glucose Levels Since Birth?

**DOI:** 10.4274/jcrpe.galenos.2018.2018.0141

**Published:** 2019-05-28

**Authors:** Citlalli E. Hernández-Rodríguez, Cynthia M. Estrada Zúñiga, Manuel E. De la O-Cavazos, Fernando F. Montes-Tapia, Patricia Gerez-Martínez, Fernando J. Lavalle-González, Consuelo Treviño Garza

**Affiliations:** 1 Universidad Autonoma de Nuevo Leon, Facultad de Medicina y Hospital Univeristario “Dr. Jose E. Gonzalez”, Department of Pediatrics, Monterrey, Mexico; 2 Universidad Autonoma de Nuevo Leon, Facultad de Medicina y Hospital Univeristario “Dr. Jose E. Gonzalez”, Department of Endocrinology, Monterrey, Mexico

**Keywords:** Vaspin, insulin, glucose, birth weight, cord blood

## Abstract

**Objective:**

Hormones produced by fat tissue, adipokines, produced during intrauterine life have recently been implicated in fetal growth. Vaspin is an adipokine expressed in visceral adipose tissue and has insulin-sensitizing effects. Elevated serum vaspin concentrations are associated with alterations in insulin sensitivity. We aimed to determine if vaspin concentrations in cord blood from healthy, term newborns differ among those born small for gestational age (SGA), appropriate for gestational age (AGA), and large for gestational age (LGA). A secondary objective was to determine whether an association existed between vaspin and anthropometric measurements, glucose and insulin levels in the newborn.

**Methods:**

The study population included healthy term newborns, 30 subjects in the SGA, 12 in the AGA, and 34 in the LGA group. Anthropometry was documented in all subjects. Blood was taken from the umbilical cord vein from each child for later analysis for vaspin, insulin and glucose concentrations.

**Results:**

Cord blood vaspin, insulin and glucose concentrations were not different between the three study groups. A negative correlation between vaspin and glucose concentrations was demonstrated in the whole cohort (r=-0.364, p=0.001). This correlation was also observed in the LGA group (r=-0.482, p=0.004). Glucose concentrations significantly predicted vaspin concentrations (r^2^=0.132, p=0.001).

**Conclusion:**

We found a negative association between glucose and vaspin concentrations in umbilical cord blood. In addition there was a predictive association between blood glucose and resulting vaspin concentration, suggesting that vaspin can be used as a predictor of alterations in the insulin-glucose metabolism from birth.

**What is already known on this topic?**

Vaspin, is expressed in visceral adipose tissue and has insulin-sensitizing effects. Elevated vaspin expression could represent a compensatory mechanism of insulin resistance secondary to the metabolic complications of obesity.

**What this study adds?**

The results showed negative association between glucose and vaspin levels in umbilical cord blood. The predictive nature of glucose levels on vaspin levels support the idea that elevated vaspin levels protect against insulin resistance.

## Introduction

The belief that adipose tissue was only an energy reservoir began to change with the discovery of hormones, known as adipokines, produced by fat tissue, conferring an endocrine function on fat deposits (1).

Adipokines, which are produced during intrauterine life, have recently been implicated in fetal growth. Thus there is a growing interest in exploring their physiology in early life (2). Vaspin, identified as a member of the serine protease inhibitor family, is specifically expressed in visceral adipose tissue and has insulin-sensitizing effects. Elevated vaspin concentrations in serum are associated with obesity and alterations in insulin sensitivity in humans (3), even in infancy (4).

The administration of vaspin improved glucose tolerance and insulin sensitivity in rodents. Moreover, the administration of insulin significantly upregulated vaspin mRNA in subcutaneous adipose tissue, and to a lesser extent, reduced expression in visceral adipose tissue. These authors concluded that elevated vaspin expression could represent a compensatory mechanism of insulin resistance, secondary to the metabolic complications of obesity (3,5). Therefore, better understanding of the biology of vaspin may lead to the development of new treatment strategies for obesity, diabetes and insulin resistance (6).

Alterations in fetal nutrition can result in development adaptations that permanently change the physiology and metabolism of the progeny, specifically, insulin–glucose metabolism (7). This is supported by the recognition of an increased risk of developing type 2 diabetes, hypertension and metabolic syndrome later in life in infants that are either small for gestational age (SGA) or large for gestational age (LGA) (8,9).

The objective of this study was to determine if vaspin concentrations in cord blood of healthy, term newborns differed among SGA, appropriate for gestational age (AGA) and LGA newborns. A secondary objective was to determine whether there is an association between vaspin and anthropometry and glucose and insulin concentrations in the newborn.

## Methods

The study was approved by the Ethics Committee in Research of the Hospital Universitario “Dr. Jose Eleuterio Gonzalez” with the code PE16-00013. A written informed consent before enrollment was obtained from all mothers.

Seventy-six newborns (>37 weeks gestation) evaluated from December 2012 to January 2015 at the Universidad Autonoma de Nuevo Leon Medical School and the “Dr. Jose E. Gonzalez” University Hospital in Monterrey, Mexico were included in the study.

The study population consisted of healthy term newborns. Exclusion criteria were newborns of mothers with gestational diabetes, pregestational diabetes mellitus, pre-eclampsia, hypertension or thyroid disease. Elimination criteria were any disease that required inpatient management or intercurrent disease affecting nutritional status.

A blood sample was taken from the umbilical cord vein from each infant immediately after birth and centrifuged at 1600 g and 4 ºC. Aliquots of serum were separated, frozen, and stored at -70 °C for later analysis for vaspin, insulin and glucose. Birth weight was documented prospectively using a Torrey scale (Torrey, S.A. de C.V., Monterrey, Mexico) and length was obtained using a SECA 210 infantometer (SECA North America, Chino, CA., USA) at birth. The total sample was classified into three groups, SGA (less than 10th percentile), AGA (percentile between 10 and 90) or LGA (greater than 90^th^ percentile), according to birth weight for gestational age (10).

Serum vaspin concentrations were analyzed by enzyme-linked immunosorbent assay (ELISA) using a commercial kit (Biovendor Human Vaspin ELISA, Brno, Czech Republic) according to the manufacturer’s instructions. Sensitivity of the assay was 0.01 ng/mL, the intra- and interassay coefficients of variation were 7.6% and 7.7%, respectively. The values reported below the sensitivity of the assay reported as <0.010 ng/nL were scaled to 0.010 ng/mL.

The determination of insulin concentrations was performed by electrochemiluminescence immunoassay using a commercial kit (Roche Diagnostics, Indianapolis, IN., USA). Sensitivity of the assay was 0.2 μU/mL; the intra- and interassay coefficient of variation were 3.6% and 3.4%, respectively. Glucose concentrations were determined by the glucose oxidase method using a comercial kit (Pointe Scientific Inc., Canton, MI., USA) according to the manufacturer’s instructions.

### Statistical Analysis

Measures of central tendency are presented as medians (range) and means ± standard deviation, according to the distribution of the variables.

The Kolmogorov-Smirnov test was applied to check the normality of the variables. A non-Gaussian distribution was shown for the data of vaspin and insulin, while glucose data were normally distributed.

The chi-square test was used to compare proportions. For comparison of dimensional continuous variables, a non-parametric Mann-Whitney U test and Kruskal-Wallis test were performed. Univariate analysis of variance was used for normally distributed data.

Pearson’s correlation coefficient was applied to detect any positive or negative correlations. For multiple regression analysis, the stepwise forward model was used. A p ≤0.05 was considered statistically significant. Statistical Package for the Social Sciences for Macintosh, v.22.0 (IBM Corp., Armonk, NY., USA) was used for analysis.

## Results

The study population (n=76) included 30 subjects in the SGA group, 12 in the AGA group and 34 in the LGA group. Demographic and anthropometric characteristics are presented in Table 1. None of the groups showed significant differences in terms of gender, delivery route, gestational age or mother’s age.

Cord blood-derived serum vaspin, insulin and glucose concentrations were not different among the three study groups; concentrations and comparisons are shown in Table 2. A significant correlation between vaspin concentration and birth length (cm) was found (r=0.277, p=0.016), but not with birth weight or cephalic perimeter (see Table 3).

**Figure 1:**
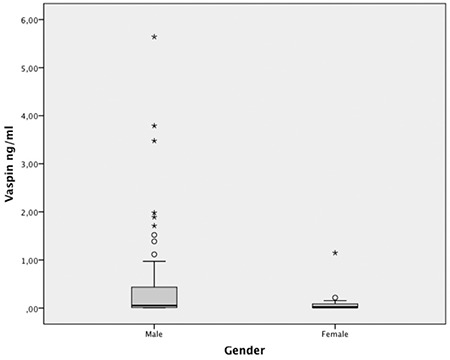
Serum vaspin concentrations in male (n=44) and female (n=32) newborns

Median (range) cord blood vaspin concentrations were significantly higher in males, 0.054 (0.010-5.64) ng/mL compared to females, 0.017 (0.010-1.14) ng/mL across the whole cohort (p=0.034) (Figure 1). Insulin correlated with birthweight in the total population (r=0.328, p=0.004; see Table 3). However when this was analyzed by study group, the correlation was only present in the LGA group (r=0.507, p=0.002). Likewise, a positive correlation was found between insulin and glucose only in the SGA group (r=0.400, p=0.028). A negative correlation between vaspin and glucose concentrations was demonstrated in the total population (r=-0.364, p=0.001; see Table 3). In the analysis by study group, this correlation was only observed in the LGA group (r=-0.482, p=0.004).

Birth length weakly, but significantly, predicted vaspin cord blood concentrations for the total population (r^2^=0.077, p=0.016). In the multivariate analysis, including either a stepwise or an all-at-once approach, the anthropometric variables (birth weight and cephalic perimeter) did not increase prediction of vaspin levels (Table 4).

Glucose levels significantly predicted vaspin levels (r^2^=0.132, p=0.001). In the multivariate analysis, including either a stepwise or an all-at-once approach, including insulin in the model did not increase the prediction of vaspin levels (Table 4).

## Discussion

Vaspin, which has been recently discovered, and with promising beneficial effects on obesity and diseases related to insulin resistance, could be the basis for future pharmacological treatment (11). However the biology and metabolism of vaspin has not yet been extensively studied. To understand the role of vaspin in metabolically important periods, such as fetal life, is of great importance.

This study included a sample of healthy term newborns of mothers without any diagnosed comorbidities that could have interfered with normal weight gain during intrauterine life. No differences in vaspin concentrations in umbilical cord serum were found between the SGA, AGA and LGA study groups. Vaspin concentrations were significantly higher in males than females. It was also found that length at birth and glucose concentration were independent variables that predicted vaspin concentration in umbilical cord blood.

In a previous study, Akcay et al (12) reported higher vaspin levels in the SGA group when compared to the AGA and LGA groups, concluding that this finding may be the result of differences in energy homeostasis in intrauterine life, since the SGA group had a reduced fat mass, an altered development of adipose tissue and relatively higher visceral fat deposits. The authors suggested that greater visceral fat deposits may be the source of higher vaspin concentrations in SGA neonates (9,13).

Human vaspin concentrations have been reported to be associated with obesity, insulin resistance and type 2 diabetes mellitus type 2 (14,15). Kafalidis et al (16) reported higher vaspin levels in an LGA group when compared with AGA infants, attributing these differences to altered fat accumulation and hyperinsulinemia in the LGA group.

A previous study compared vaspin concentrations in umbilical cord blood of newborns with intrauterine growth restriction (IUGR) and AGA without reporting statistically significant differences (17). Cekmez et al (18) studied vaspin levels in LGA and AGA and reported no statistically significant difference between the two study groups. The absence of differences in the previous reports, similar to this report, might be due to differences in race, or differences in the definition of SGA or LGA.

Although it has been previously described that vaspin can play a major role in fetal development (19), published studies do not report an association between cord blood vaspin concentrations and length at birth, as was found in this study. It is known that fetal macrosomia is related to hyperinsulinemia during fetal development, which is a result of elevated maternal glucose concentrations which allows glucose to be transferred across the placenta, and further concentrations produced by the fetal pancreas during the second trimester, when insulin is secreted autonomously and independently of maternal glucose stimulation (Pedersen’s hypothesis) (20). It is possible that this mechanism leads to increased vaspin production to improve utilization of insulin, thus reducing glucose levels, to achieve an optimal intrauterine environment. However, this mechanism needs to be studied more extensively, since in our study vaspin levels were positively correlated with length at birth.

Körner et al (21) reported significantly higher serum vaspin concentrations in girls than in boys at ages seven to 18 years. They showed an increase in female vaspin levels at puberty while a non-dynamic increase was found in vaspin at puberty in boys. Consequently, it appears that the greatest difference between girls and boys occurs in the adolescent age group, despite a lack of correlation between sex steroids (estradiol and testosterone) and vaspin levels.

Briana et al (17) also reported higher vaspin levels in females than in males in a IUGR sample on postnatal day 1, while no difference was observed in umbilical cord blood. In contrast Akcay et al (12) did not find a difference by gender in vaspin levels in umbilical cord blood, despite differences in adipose tissue distribution and mass. In our study, in contrast to these previous studies, we found significantly elevated cord blood vaspin levels in males compared to females. Gender dependent behavior has been demonstrated for adiponectin (22) and leptin (23). It is not known if the same is true for vaspin and this remains to be elucidated as current evidence is contradictory.

Klöting et al (14) have reported that a single intracerebroventricular injection of vaspin was sufficient to cause a sustained and significant improvement in glucose concentration over at least six days in *db/db* mice, which are a rodent model of insulin resistance, but not in C57BL/6 mice. The authors suggest that these results indicate that vaspin reduces plasma glucose only in the presence of elevated blood glucose concentrations and go on to suggest that treatment with vaspin would not have the potential to cause hypoglycemia. In reference to our results, this mechanism may explain the negative correlation between vaspin and glucose concentrations which were only observed in the LGA group. It can be hypothesized that this group developed in an abnormal intrauterine environment, reflecting mild maternal hyperglycemia below the diagnostic threshold. Evidence to support this hypothesis comes from Chiesa et al (24) who reported that even a limited degree of maternal hyperglycemia, even within the normal range, may affect fetal weight. This finding is also supported by Hida et al (3) who administered vaspin to diet-induced obese mice, which significantly improved insulin sensitivity and glucose tolerance, while administration of vaspin to *in vivo* lean mice did not alter glucose tolerance. The authors concluded that the upregulation of vaspin may be a protective mechanism for insulin resistance.

The predictive nature of cord blood glucose concentration for vaspin cord blood concentration reflects their interdependence. Fetal vaspin concentration is increased in response to elevated glucose, possibly from maternal circulation. As vaspin improves insulin resistance an increase in concentration will have the effect of improved fetal insulin utilization. This appears to be a compensatory mechanism for reducing fetal glucose, possibly derived from maternal sources, in order to achieve an optimal intrauterine environment.

### Study Limitations

The main limitation of the study is the characteristics of the sample obtained for convenience, also, we did not have another study group of mothers with gestational diabetes, because it would allow comparison of the LGA group of mothers without co-morbidities and those with a clear alteration of the insulin-glucose metabolism. In addition, we did not evaluate the maternal vaspin and glucose concentrations and thus we can not have direct evidence of the relationship between maternal hyperglycemia resulting in LGA newborns with elevated vaspin levels.

However, the fact of having a sample without comorbidities allows us to conclude that birth weight determines alterations in the insulin-glucose metabolism where vaspin can serve as a marker.

## Conclusion

We found a negative association between glucose and vaspin levels in umbilical cord blood. In addition glucose levels were found to be predictive of vaspin levels, supporting the idea that elevated vaspin levels may have a protective action against insulin resistance in the intrauterine period and suggesting that vaspin may be used as a predictor of alterations in insulin-glucose metabolism. This may be especially true in target populations, such as the LGA group. Further studies are needed to investigate the role of vaspin in newborns of mothers with a history of insulin resistance to confirm its involvement in pathological processes.

## Figures and Tables

**Table 1:**
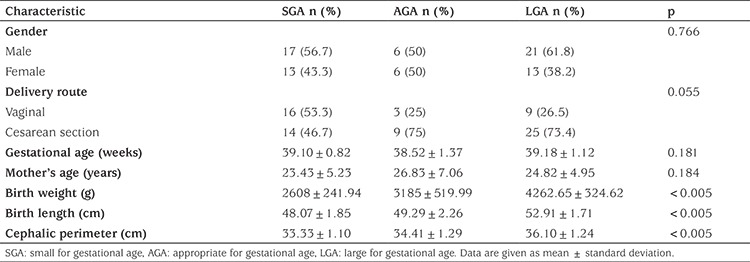
Demographic and anthropometric characteristics of the study groups

**Table 2:**
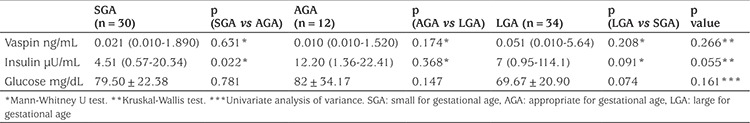
Vaspin, insulin and glucose concentrations by study groups

**Table 3:**
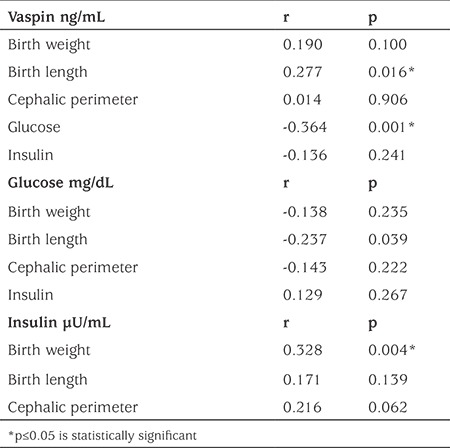
Correlations of vaspin, glucose and insulin with one another and with anthropometric variables in the total population

**Table 4:**
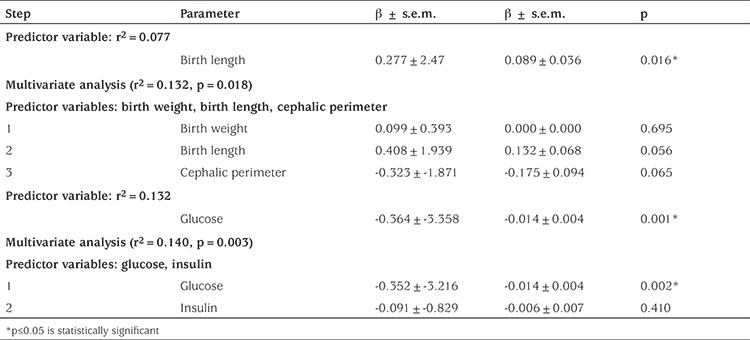
Predictor variables and multivariate analysis using vaspin as a dependent variable
